# Risk Factors for Noninvasive Ventilation Failure in Children Post-Hematopoietic Cell Transplant

**DOI:** 10.3389/fonc.2021.653607

**Published:** 2021-05-27

**Authors:** Courtney M. Rowan, Julie C. Fitzgerald, Asya Agulnik, Matt S. Zinter, Matthew P. Sharron, James E. Slaven, Erin M. Kreml, Rajinder P.S. Bajwa, Kris M. Mahadeo, Jerelyn Moffet, Keiko M. Tarquinio, Marie E. Steiner

**Affiliations:** ^1^ Department of Pediatrics, Division of Critical Care, Riley Hospital for Children, Indiana University School of Medicine, Indianapolis, IN, United States; ^2^ Department of Anesthesiology and Critical Care, Children’s Hospital of Philadelphia, University of Pennsylvania Perelman School of Medicine, Philadelphia, PA, United States; ^3^ Department of Global Pediatric Medicine, Division of Critical Care, St. Jude’s Children’s Research Hospital, Memphis, TN, United States; ^4^ Department of Pediatrics, Division of Critical Care, University of California San Francisco, San Francisco, CA, United States; ^5^ Department of Pediatrics, Division of Critical Care, George Washington University School of Medicine and Health Sciences, Children’s National, Washington, DC, United States; ^6^ Department of Biostatistics, Indiana University School of Medicine, Indianapolis, IN, United States; ^7^ Critical Care Medicine, Phoenix Children’s Hospital, Phoenix, AZ, United States; ^8^ Division of Heme/Onc/Bone Marrow Transplant, Nationwide Children’s Hospital, Columbus, OH, United States; ^9^ Department of Pediatrics, Division of Pediatric Stem Cell Transplant and Cellular Therapy, University of Texas at MD Anderson Cancer Center, Houston, TX, United States; ^10^ Department of Pediatrics, Division of Blood and Marrow Transplant, Duke Children’s Hospital, Duke University, Durham, NC, United States; ^11^ Department of Pediatrics, Division of Pediatric Critical Care Medicine, Emory University, Children’s Healthcare of Atlanta, Atlanta, GA, United States; ^12^ Department of Pediatrics, Division of Critical Care and Division of Hematology, Masonic Children’s Hospital, University of Minnesota, Minneapolis, MN, United States

**Keywords:** hematopoietic (stem) cell transplantation (HCT), noninvasive (positive pressure) ventilation, respiratory insufficiency, intubation, cardiopulmonary resuscitation

## Abstract

**Rationale:**

Little is known on the use of noninvasive ventilation (NIPPV) in pediatric hematopoietic cell transplant (HCT) patients.

**Objective:**

We sought to describe the landscape of NIPPV use and to identify risk factors for failure to inform future investigation or quality improvement.

**Methods:**

This is a multicenter, retrospective observational cohort of 153 consecutive children post-HCT requiring NIPPV from 2010-2016.

**Results:**

97 (63%) failed NIPPV. Factors associated with failure on univariate analysis included: longer oxygen use prior to NIPPV (p=0.04), vasoactive agent use (p<0.001), and higher respiratory rate at multiple hours of NIPPV use (1hr p=0.02, 2hr p=0.04, 4hr p=0.008, 8hr p=0.002). Using respiratory rate at 4 hours a multivariable model was constructed. This model demonstrated high ability to discriminate NIPPV failure (AUC=0.794) with the following results: respiratory rate >40 at 4 hours [aOR=6.3 9(95% CI: 2.4, 16.4), p<0.001] and vasoactive use [aOR=4.9 (95% CI: 1.9, 13.1), p=0.001]. Of note, 11 patients had a cardiac arrest during intubation (11%) and 3 others arrested prior to intubation. These 14 patients were closer to HCT [14 days (IQR:4, 73) vs 54 (IQR:21,117), p<0.01] and there was a trend toward beginning NIPPV outside of the PICU and arrest during/prior to intubation (p=0.056).

**Conclusions:**

In this cohort respiratory rate at 4 hours and vasoactive use are independent risk factors of NIPPV failure. An objective model to predict which children may benefit from a trial of NIPPV, may also inform the timing of both NIPPV initiation and uncomplicated intubation.

## Introduction

In children, noninvasive positive pressure ventilation (NIPPV) practices are extrapolated from adult literature or based on evidence from small retrospective pediatric studies. NIPPV use is not standardized in pediatric critical care and reported success rates are variable. In critically ill children, NIPPV may decrease the rates of intubation (1–3). Timing plays an important role as studies suggest that those who respond to NIPPV do so early in the course of respiratory illness (1, 4).

Children post-allogeneic hematopoietic cell transplant (HCT) who need invasive mechanical ventilation have a mortality of 60% (5). Means to prevent, but not delay, intubation would be useful particularly in this population. NIPPV seems to be a logical step in prevention of progression of respiratory failure. However, optimal application of this modality remains elusive. Factors predicting progression of respiratory, or NIPPV failure, resulting in intubation are unknown in children post-HCT. In adult trials of NIPPV use in immunocompromised patients, there are conflicting data regarding oxygenation, intubation rates, and mortality (6, 7). One study in the pediatric oncology population found a failure rate of 25% but included few stem cell transplant patients (8). Furthermore, there is questionable success of NIPPV use in acute respiratory distress syndrome (ARDS) (9, 10) which is common in HCT patients with respiratory failure (11).

Several studies have reported predictors of NIPPV failure in children. Higher acuity scores, multiorgan dysfunction, shock, sepsis, severe hypoxia, ARDS, respiratory acidosis, and oncologic diagnosis have all demonstrated association with NIPPV failure (4, 12–14). Given the suggestion that NIPPV fails in those with underlying oncology diagnosis and ARDS, we sought to investigate the use of NIPPV in the pediatric allogeneic HCT patient and to identify factors associated with NIPPV failure.

## Materials and Methods

We conducted a multicenter, retrospective, cohort study of all consecutive children and young adults, aged 1 month to 21 years, who were treated with NIPPV following an allogeneic HCT. Eleven centers (**Supplementary Table 1**) participated. Regulatory approval was obtained at each center. Patients were included in the study if they received NIPPV between 2010 and 2016 and were within 1 year post-allogeneic HCT. NIPPV was defined as either continuous positive airway pressure or bilevel positive airway pressure (BIPAP). Patients were excluded if NIPPV was only used post-extubation or if the patient had limitations on life-sustaining measures including intubation or resuscitation at initiation of NIPPV. If the patient received multiple courses of NIPPV, only the first course of NIPPV was included. Failure of NIPPV was defined as either 1) needing intubation for critical illness (i.e. excluding intubation for procedures), or 2) a cardiopulmonary arrest prior to intubation. NIPPV success was defined as being weaned off NIPPV for a minimum of 24 hours without meeting failure criteria or back to baseline respiratory support (i.e. nighttime NIPPV).

Patients were identified by review of institutional health records. Abstracted data included demographics; HCT characteristics; day post-HCT at NIPPV initiation; the clinical diagnosis of graft vs. host disease and/or veno-occlusive disease prior to/during NIPPV use. Daily white blood cell count was collected at NIPPV initiation. Length of supplemental oxygen support prior to NIPPV, change in weight from admission to NIPPV initiation, use of high flow nasal cannula prior to NIPPV, location where NIPPV was started, cause of respiratory distress, use of vasoactive agents, and use of continuous renal replacement therapy (CRRT) were also collected. Vasoactive use was defined as the use of any continuous infusion of vasoactive agents up to and including the day of intubation. CRRT use was noted up to and including day of intubation. Vital signs, weight, fluid balance and respiratory support parameters were collected at multiple *a priori* defined time points through the first 3 days or up to failure of NIPPV, if this occurred before 3 days. The SpO_2_/FiO_2_ was calculated however, the analysis was not included due to a significant portion of missing/valid data (71% missing/unable to calculate), mostly secondary to the SpO_2_ being > 97%. Documented complications attributed to NIPPV were collected. The primary outcome was NIPPV failure. PICU mortality, hospital mortality, length of PICU stay, length of hospital stay, cardiopulmonary arrest during intubation, and cardiac arrest at any time point in the PICU were secondary outcomes. Data was stored in single Research Electronic Data Capture database at Indiana University (15).

### Statistical Methods

Medians and interquartile ranges for continuous variables and frequencies and percentages for categorical variables were used to describe the cohort. Standard inferential statistics compared patients with a successful NIPPV course to those who failed NIPPV, with Chi-Square tests being used for categorical variables (Fisher’s Exact when expected cell counts were small) and Wilcoxon rank sum tests for continuous variables. The primary analysis focused on determining factors associated with NIPPV failure. Bivariate and multivariable logistic regression measured the association of potential risk factors with the primary outcome. Variables included in the multivariate model were those that had a p value < 0.05 in bivariate analyses. *A priori*, age was determined to be part of the model regardless of bivariable association due to the age effect on various vital signs investigated. Goodness of fit for the multivariable model was determined with the Hosmer and Lemeshow test, and area under the curve (AUC) was calculated. Center effect was explored with a mixed effect model with center being treated as a random effect. Center was not associated with failure (p=0.731) and did not change the significance of any of the other variables investigated.

Respiratory rate was categorized into high/low determined by a receiver operating characteristics curve and Youden’s index. Further strengthening the decision to use a single cutpoint, multiple sensitivity analyses were run using different age groups. Using Youden’s index for individual cut-points for various age groupings, the respiratory rate cut-point identified was consistently between 35 to 42 breaths per minute regardless of the way age groups were categorized (**Supplementary Table 2**). Analyses were performed using SAS v9.4 statistical software (SAS Institute, Cary, NC).

## Results

Of the 153 patients included in analysis, 63% (n=97) failed NIPPV. The median age of the cohort was 11 (IQR: 3-15) years and 46% were female. Those who were successfully treated NIPPV were more likely to have a matched related donor than those who failed, otherwise there were no demographic differences (**Table 1**). Details of specific diagnosis leading to transplant can be found in **Supplementary Table 3**. Those successfully treated with NIPPV had a longer median length of NIPPV use compared to those who failed [2.1 (IQR:0.8, 4.1) vs 0.7 (IQR: 0.2, 2.4) days, p< 0.001]. The time to NIPPV failure was variable with the majority occurring within the first 48 hours of NIPPV initiation (**Supplementary Figure 2**). The median time to NIPPV failure was 0.7 (IQR: 0.2, 2.4) days.

**Table 1:** Patient characteristics stratified by failure or successful use of noninvasive positive pressure ventilation.

	Entire cohortn=153	NIPPV failuren=97	NIPPV successn=56	p-value
Age in years	10.7 (3.4, 14.8)	9.8 (3.3, 14.3)	12.1 (4.2, 15.5)	0.28
Gender (female)	70 (45.8)	42 (43.3)	28 (50.0)	0.42
Weight (kg) at hospital admission	30.0 (15.5, 54.6)	29.6 (14.9, 49.7)	38.6 (16.6, 55.0)	0.39
1^st^ Transplant	126 (82.9)	80 (83.3)	46 (82.1)	0.85
Source of Transplant Bone Marrow Cord Blood PBSC	68 (44.4) 51 (33.3) 34 (22.2)	38 (39.2) 38 (39.2) 21 (21.7)	30 (53.6) 13 (23.2) 13 (23.2)	0.11
Matched related donor^a^	27 (17.7)	11 (11.3)	16 (28.6)	0.009
Conditioning Myeloablative Reduced intensity Nonmyeloablative	103 (67.3) 38 (24.8) 12 (7.8)	64 (66.0) 23 (23.7) 10 (10.3)	39 (69.6) 15 (26.8) 2 (3.6)	0.37
Malignant diagnosis leading to transplant	83 (54.2)	56 (57.7)	27 (48.2)	0.23
Days post HCT at start of NIPPV	44.5 (16.4, 118.3)	51.5 (19.5, 110.5)	44.2 (14.8, 139.8)	0.94
WBC count at start of NIPPV (x 10^3^/mm^3^)	2.7 (0.4, 6.8)	2.4 (0.3, 7.6)	3.1 (0.7, 5.5)	0.51
Graft vs. host disease^b^	53 (34.6)	37 (38.1)	16 (28.6)	0.26
Veno-occlusive disease^b^	22 (14.8)	13 (13.4)	9 (16.1)	0.64

Values are medians (IQRs) for continuous variables and frequencies (percentages) for categorical variables, with p-values from Wilcoxon Rank Sum and Chi-Square (Fisher’s Exact when cell counts were small) tests, respectively. P values for conditioning regimen and source of transplant demonstrate among the three variables difference for those that failed NIPPV to those that were successfully treated. Since the overall p was not significant, correction for multiple comparison was not utilized.

^a^Matched related donor was compared to all other donor types including mismatched related donor, matched unrelated donor, unrelated, mismatched donor.

^b^Diagnosed prior to or while on NIPPV.

NIPPV, noninvasive positive pressure ventilation; PBSC, peripheral blood stem cells; PICU, pediatric intensive care unit; HCT, hematopoietic cell transplant; WBC, white blood cell count.

### Initiation of NIPPV

Patient characteristics at time of initiation of NIPPV are found in **Table 2**. The cause of respiratory distress was variable. The most common reason was a pulmonary infection (39.2%), which was more likely in those who failed NIPPV (p=0.05). The majority (77.8%) of NIPPV was initiated in the PICU. Those who failed spent a longer time on supplemental oxygen prior to NIPPV initiation (p=0.038). Over 40% of patients were treated with high flow nasal cannula prior to NIPPV and this was not associated with NIPPV failure. The most common type of NIPPV utilized was BIPAP, with 75% of the cohort receiving this at initiation of NIPPV. Initial type of NIPPV was not associated with failure.

**Table 2:** Patient characteristics at NIPPV initiation stratified by failure or successful use of noninvasive positive pressure ventilation.

	Entire cohortn=153	NIPPV failuren=97	NIPPV successn=56	p-value
*Reasons for respiratory failure:*				
Respiratory infection/Pneumonia	60 (39.2)	44 (45.4)	16 (28.6)	0.05
Septic Shock	32 (20.9)	20 (20.6)	12 (21.4)	0.92
Fluid overload/pulmonary edema/pleural effusions	28 (18.3)	15 (15.5)	13 (23.2)	0.23
Hypoxia of unknown origin	15 (9.8)	8 (8.2)	7 (12.5)	0.39
Upper airway obstruction	11 (7.2)	8 (8.2)	3 (5.4)	0.54
Pulmonary hemorrhage/Hemoptysis	10 (6.5)	7 (7.2)	3 (5.4)	0.75
Altered Mental status	9 (5.8)	5 (5.2)	4 (7.1)	0.73
IPS/engraftment syndrome/pulmonary GVHD	9 (5.8)	5 (5.2)	4 (7.1)	0.73
Other	3 (2.0)	3 (3.1)	0 (0.0)	0.30
*Place NIPPV started*				0.99
PICU	119 (77.8)	76 (78.4)	43 (76.8)	
HCT ward	32 (20.9)	20 (20.6)	12 (21.4)	
Emergency Room	2 (1.3)	1 (1.0)	1 (1.8)	
Days on supplemental oxygen prior to the initiation of NIPPV	2.0 (1.0, 6.0)	3.0 (1.0, 6.0)	1.0 (1.0, 4.5)	0.038
Use of high flow nasal cannula prior to initiation of NIPPV	62 (40.5)	43 (44.3)	19 (33.9)	0.181
*Starting type of NIPPV (n=150)^a^*				0.579
BIPAP	111 (74.0)	71 (75.5)	40 (71.4)	
CPAP	39 (26.0)	23 (24.5)	16 (26.8)	
*Starting settings of NIPPV*				
Inspiratory pressure (cm H_2_0)	10 (6, 10)	10 (6, 12)	12 (7, 14)	0.118
Expiratory pressure (cm H_2_0)	6 (6, 8)	6 (6, 8)	6 (6, 8)	0.848
Vasoactive use^b^	53 (34.6)	43 (44.3)	10 (17.9)	0.0001
CRRT prior to intubation	21 (13.8)	17 (17.5)	4 (7.3)	0.078

Values are medians (IQRs) for continuous variables and frequencies (percentages) for categorical variables, with p-values from Wilcoxon Rank Sum and Chi-Square (Fisher’s Exact when cell counts were small) tests, respectively. Subjects often had more than one cause leading to respiratory failure, therefore total does not =100%.

^a^3 patient were missing detailed data on settings of NIPPV at initiation.

^b^Vasoactive use was defined as the use of any continuous infusion of vasoactive agents up to and including the day of intubation.

NIPPV, noninvasive positive pressure ventilation; IPS, idiopathic pneumonia syndrome; GVHD, graft vs host disease; PICU, pediatric intensive care unit; HCT, hematopoietic cell transplant; BIPAP, Bilevel positive airway pressure; CPAP, continuous positive airway pressure; CRRT, continuous renal replacement therapy.

### Risk Factors for NIPPV failure

We examined multiple vital signs and respiratory parameters including NIPPV settings, FiO2, heart rate, blood pressure, SpO_2_, and respiratory rate at NIPPV initiation, 2, 4, 8, and 12 hours after initiation (**Supplementary Table 4**). Of these variables, we found a higher respiratory rate was associated with NIPPV failure at multiple time points (Initiation p= 0.03, 2 hour p=0.045, 4 hour p=0.005, 8 hour p=0.003, 12 hour p=0.002). Median respiratory rate was consistently higher in those that failed NIPPV (**Supplementary Figure 2**). We focused on the respiratory rate at 4 hours because we were aiming for early risk factors and this time point had the most complete data, minimizing missingness in the model. Evaluating respiratory rate at 4 hours as a continuous variable, we found that the odds of failure increased with higher respiratory rate. For every 10 -breaths/minute increase in respiratory rate the odds of NIPPV failure increased: OR=1.4 (95%CI: 1.1, 1.7), p=0.008. The remainder of the variables examined were not significantly associated with NIPPV failure.

To improve the clinical applicability of the model, respiratory rate was evaluated as a categorical variable. As age was not different between the two groups, and the majority of the patients in this cohort were school-aged, a single cut-point was chosen. Using the cohort as a whole, a respiratory rate of 40 was identified as the optimal cut-point. A higher percentage of patients who failed NIPPV had a respiratory rate > 40 at hour 4 of NIPPV, p< 0.001 (**Supplementary Figure 3**), and, in univariable logistic regression analysis, a respiratory rate > 40 was associated with an increased risk of NIPPV failure, OR=3.8, (95% CI: 1.7, 8.6, p=0.001).

In addition to the vital signs and respiratory parameters evaluated in **Supplementary Table 4**, other variables were assessed to determine their association with NIPPV failure. Variables of interest included days on supplemental O_2_ prior to NIPPV, vasoactive agent use, ≥ 10% and ≥15% weight gain from hospital admission to NIPPV initiation, and the use of CRRT prior to NIPPV failure. These univariate analyses are presented in **Supplementary Table 5**. Of these variables, vasoactive agent use and supplemental O_2_ prior to NIPPV were significantly associated with NIPPV failure (p=0.0001).

### Multivariate Assessment of Risk Factors for NIPPV Failure

A multivariate model was constructed including age, respiratory rate > 40 breaths-per-minute at 4 hours, vasoactive use, days of O_2_ use prior to NIPPV, and matched, related donor vs other donor types. Adjusting for these covariates, we found that RR > 40 and the use of vasoactive agents prior to/same day as NIPPV were significantly associated with an increased risk of failing NIPPV. Having a matched related donor was associated with NIPPV success (**Table 3**). This model had an area under the curve of 0.794 for predicting NIPPV failure. The positive predictive value of this model was 76% and the negative predictive value was 70%. Of note, the model was also significant with respiratory rate measured as a continuous variable (**Supplementary Table 6**), however we present the categorical respiratory rate to improve clinical utility. As 40 breaths per minute is high for older patients, and additional analysis using 30 breaths per minutes was also conducted. This model found similar results and is presented in the supplement (**Supplementary Table 7**). Similar results were also seen using continuous respiratory rate at two hours and are presented in the supplement (**Supplementary Table 8**).

**Table 3:** Multivariate model for the association of patient characteristics with NIPPV failure.

	Unadjusted OR, (95% CI)	Adjusted OR^a^ (95% CI)
RR > 40 at 4 hours	3.8 (95% CI: 1.7, 8.7), p< 0.001	6.3 (95% CI: 2.4, 16.4), p<0.001
Vasoactive use^b^	3.7 (95%CI: 1.7, 8.1), p=0.001	4.9 (95% CI: 1.9, 13.1), p=0.001
Matched related donor^c^	0.3 (95%CI: 0.1, 0.8), p=0.009	0.3 (95%CI: 0.1, 0.9), p=0.031
Area under the curve	NA	0.794

OR, odds ratio; CI, confidence interval; RR, respiratory rate in breaths per minute.

^a^Adjusted for Age and days of O2 prior to NIPPV which were not significant in this model.

^b^Vasoactive use was defined as the use of any continuous infusion of vasoactive agents up to and including the day of intubation.

^c^Matched related donor was compared to all other donor types including matched unrelated, unmatched related, and unmatched unrelated.

### NIPPV Complications

With the exception of cardiopulmonary resuscitation (CPR), few complications were noted with the use of NIPPV and development of these complications was not associated with failure (**Supplementary Table 9**). The most common complication was need for CPR, described in detail below. Skin breakdown was the next most common complication documented in 5%, followed by agitation/intolerance of NIPPV in 4%. Two patients had emesis and one had epistaxis.

### Receipt of Cardiopulmonary Resuscitation (CPR)

Twenty-six patients received CPR at some point during their PICU course (**Table 4**). Of these, three patients received CPR prior to intubation, and 11 during intubation. In this cohort with 97 NIPPV failures and 11 arrests during intubation, over 10% of the intubations were complicated by arrest. Of the patients that arrested during intubation, only 18% survived to PICU discharge. All three patients who never had an intubation attempt prior to arrest died. A subanalysis comparing these 14 patients to those that were intubated without arrest revealed they were closer to their transplant (14 vs 54 days, p<0.01). Those who arrested had a higher median respiratory rate at 4 hours [48 (IQR: 27.5, 69.0) vs 38.5 (IQR: 25.2, 46.8), p=0.196], a higher use of vasoactive agents (50% vs 43%, p=0.63) and were on supplemental O2 longer (5.0 days vs 2.5 days, p=0.20), but none of these reached statistical significance. For those that arrested during intubation, there was a trend toward being started on NIPPV outside of the PICU. Of those that failed NIPPV, 21 patients were started on NIPPV outside of the ICU (20 on the transplant unit and 1 in the emergency department). Of these 21, 24% (n=5) arrested during intubation compared to 8% (n=6) arrest rate for those that were started on NIPPV in the PICU (p=0.056).

**Table 4:** Critical care interventions and outcomes of the cohort assessed by NIPPV failure status.

	Entire cohortn=153	NIPPV failuren=97	NIPPV successn=56	p-value
Length of vasoactive use (days)	2.0 (0.8, 8.1)	1.9 (0.7, 11.2)	3.1 (1.0, 6.3)	0.846
CRRT at any time in PICU	41 (27.0)	37 (38.1)	4 (7.3)	<0.0001
Length of CRRT (days)	8.5 (3.3, 15.9)	9.1 (2.4, 15.9)	4.1 (3.8, 14.6)	0.777
Length of PICU stay (days)	10.0 (4.0, 26.0)	18.0 (7.0, 35.0)	4.0 (3.0, 7.0)	<.0001
Length of NIPPV (days)	1.0 (0.4, 3.5)	0.7 (0.2, 2.3)	2.1 (0.8, 4.0)	0.0004
Length of invasive mechanical ventilation (days)	6.8 (3.0, 17.5)	6.8 (3.0, 17.5)	n/a	n/a
CPR	26 (17.5)	26 (27.7)	0 (0)	<.0001
PICU survival	83 (54.6)	32 (33.0)	51 (92.7)	<.0001
Hospital survival	66 (43.1)	27 (27.8)	39 (69.6)	<.0001

Values are medians (IQRs) for continuous variables and frequencies (percentages) for categorical variables, with p-values from Wilcoxon Rank Sum and Chi-Square (Fisher’s Exact when cell counts were small) tests, respectively.

NIPPV, noninvasive positive pressure ventilation; CRRT, Continuous renal replacement therapy; PICU, Pediatric intensive care unit; CPR, cardiopulmonary resuscitation.

### Mortality, Morbidity and Length of Stay

**Figure 1:**
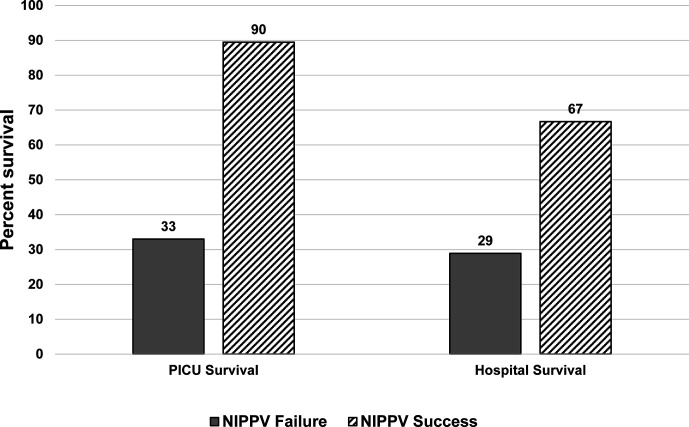
Survival assessed by NIPPV success or failure at PICU and hospital discharge. Those who failed NIPPV, represented by the solid dark gray bars had a poor PICU and hospital survival. Those who were successfully treated with NIPPV, represented by the diagonal black lines, had a high PICU survival but there was a decrease in hospital survival noted.

Those that failed NIPPV had worse outcomes (**Table 4**). They use more CRRT, had a longer length of PICU stay, and lower PICU and hospital survival. Interestingly, patients successfully treated with NIPPV and survived PICU had a sharp decline in survival to hospital discharge (**Figure 1**).

## Discussion

In this cohort of 153 children post-allogeneic HCT who used NIPPV from centers across the United States, failure of NIPPV was common and associated with significant mortality. Higher respiratory rate and receipt of vasoactive infusions prior to intubation were independent risk factors for NIPPV failure. The high rate of cardiac arrest during intubation is also a cause for concern. Objective data to help guide our medical decision-making surrounding disposition and timing of intubation are needed to improve the outcomes of these high-risk patients with respiratory failure.

Over 60% of patients failed NIPPV, highlighting the need to determine risk factors for failure to limit delays in intubation for those likely to progress, and ultimately decrease severe complications such as cardiac arrest. This failure rate is exceedingly high compared to NIPPV failure rates in the general PICU population. Three general pediatric studies found NIPPV failure rates between 15.5% and 23% (13, 16, 17), a much lower failure rate than observed in our cohort. Furthermore, in a study of 239 children with cancer treated with NIPPV, only 26% failed (8). Not only does our study reaffirm that respiratory failure is different in children post-HCT, the high failure rate reveals the urgent need to identify risk factors for failure in this population.

Our analysis identified simple clinical risk factors for NIPPV failure including, respiratory rate at four hours after NIPPV initiation and requirement for vasoactive support. Respiratory rate has been associated with NIPPV failure in other general PICU studies (13, 16, 17). It is a valuable risk factor because it is readily available in all patients, is noninvasive, and does not add additional cost to care allowing it to be evaluated globally regardless of resource availability. The use of vasoactive agents was also associated with NIPPV failure, likely representing hemodynamic instability which has been shown to be associated with NIPPV failure in children with cancer (8). Comparative effectiveness studies or quality improvement initiatives may help determine if a simple model incorporating respiratory rate and vasoactive use can improve emergent intubations and outcomes.

Many studies suggest the presence of ARDS is associated with NIPPV failure (14, 17–19). Surprisingly, these markers, including SpO_2_, FiO_2_, and SpO_2_/FiO_2_ ratio were not associated with failure in this study. One challenge was the inability to calculate an accurate SpO_2_/FiO_2_ due to multiple variables with SpO_2_> 97%. Another possible explanation is that the vast majority of patients were on very high FiO_2_ early on in the course of NIPPV making it harder to discriminate those that failed. As ARDS is extremely common in children with respiratory failure post-HCT (20), it may be less helpful in differentiating NIPPV success from failure.

The high rate of cardiac arrest prior to and during intubation should encourage both critical care and HCT physicians to critically examine NIPPV practice patterns. In large, international cohorts, the reported arrest rate during pediatric intubation ranges between 0.5%-1.7% (21–23). These data come from a variety of large, tertiary, pediatric hospitals that care for medically complex and fragile children with high acuity. Therefore, having a peri-intubation cardiac arrest rate that is 10-20 times the reported arrest rate in the general PICU population necessitates an urgent call to action to better understand and address this significant problem.

Currently, there are no guidelines for NIPPV use in children post-HCT, creating highly variable practice patterns and difficulty determining its optimal use. While many required intubation, some were successfully treated. Further, successful treatment with NIPPV resulted in improved survival and shorter PICU stay suggesting some children post-HCT benefit from its use. Given the univariate association of longer duration of supplemental oxygen prior to NIPPV with NIPPV failure, we postulate that earlier intervention with NIPPV, i.e. at the first sign of hypoxia, may offer beneficial lung recruitment and potentially slow or prevent the progression of respiratory failure. However, this requires further study. The adult immunocompromised literature suggest that early use is beneficial (6, 24, 25). Early diagnostic interventions to help target therapy, early and aggressive fluid management, and collaboration between intensivists and transplant physicians are essential to optimize NIPPV use.

Early application of NIPPV, initiation in a highly monitored setting, close monitoring for subtle signs of worsening distress, and frequent reassessment of changes (or lack thereof) in vital signs in response to therapy may be starting points to tackle this issue. Objective risk scoring systems may also be useful. In both the HCT and general pediatric population, objective risk scores have been useful to early-identify risk of decompensation (26–29), however they are not specific to the successful use of NIPPV. While the starting location of NIPPV was not associated with failure, there was a concerning trend toward an increased risk of arrest during intubation for those started outside of the PICU. These data are not conclusive but should encourage a critical look at practice patterns. A highly monitored setting with intensive nursing ability, such as the PICU, may offer the optimal opportunity to implement objective risk scores and non-invasive mechanical respiratory support in these high risk patients.

While the multicenter nature of our study adds strength to this study, some limitations remain. Retrospectively, it was challenging to understand the decisions leading to timing of intubation and transfer from the transplant unit to the PICU, which are important aspects of patient care. Additionally, NIPPV use was not protocolized creating variability within and across institutions. However, while this is a limitation, it likely represents the real-world application of NIPPV across the US. We also did not have the data to collect a vaso-inotropic score which may be an important variable for consideration in future investigation. Finally, important conversations surrounding goals and end of life clearly play a significant role in the decision to intubate children with a high mortality risk. As these conversations are often poorly documented, occur within different services at different institutions, and are very complex, it is challenging to understand how they affect the decisions surrounding intubation. As these challenges are likely universal across institutions, we believe our findings are likely representative of the management and outcomes of NIPPV in pediatric HCT patients in the US.

In summary, the high rate of cardiac arrest and mortality among children post-HCT receiving NIPPV should raise a high level of concern and push our field to consider earlier intubation in this population. If NIPPV is to be optimally utilized in the HCT population, an objective model to identify which children may benefit from a trial of NIPP. It may also inform the timing of NIPPV initiation and help reduce the rates of complications with intubation. In this study we found that respiratory rate at 4 hours and vasoactive use are independent risk factors for NIPPV failure.

## Data Availability Statement

The original contributions presented in the study are included in the article/**Supplementary Material**, further inquiries can be directed to the corresponding author.

## Ethics Statement

The studies involving human participants were reviewed and approved by each participating institution’s regulatory board. Written informed consent from the participants’ legal guardian/next of kin was not required to participate in this study in accordance with the national legislation and the institutional requirements.

## Author Contributions

CR and MS were responsible for study conception and oversight. CR, JF, AA, MZ, MS, and MS were involved with study design and development of the data collection forms. JS and CR were the primary data analysts. CR, JF, AA, MZ, and MS were responsible for the primary interpretation of the results. CR, JF, AA, MZ, MS, EK, RB, KM, JM, KT, and MS provided additional data collection and interpretation of results. All authors contributed to the article and approved the submitted version. CR is the guarantor of the manuscript.

## Funding

St. Baldrick’s Foundation.

## Conflict of Interest

The authors declare that the research was conducted in the absence of any commercial or financial relationships that could be construed as a potential conflict of interest.
