# Supplementary material for: Risk Factors for Noninvasive Ventilation Failure in Children Post-Hematopoietic Cell Transplant
Source: Front Oncol. 2021 May 27;11:653607. doi: 10.3389/fonc.2021.653607 (PMC8190382; doi:10.3389/fonc.2021.653607)
Supplement: Supplementary file 1 [file Presentation_1.pdf]

Risk factors for noninvasive ventilation failure in children post-hematopoietic cellular transplant

Authors:

Courtney M, Rowan, MD, MS[1]; Julie C Fitzgerald, MD, PhD[2]; Asya Agulnik, MD, MPH[3]; Matthew S. Zinter, MD[4]; Matthew P. Sharron, MD[5]; James Slaven, MS[6]; Erin M. Kreml, MD[7]; Rajinder PS Bajwa, MD[8]; Kris Mahadeo, MD[9]; Jerelyn Moffet, NP[10]; Keiko M. Tarquinio, MD[11]; Marie Steiner, MD[12] for the Hematopoietic Cell Transplant subgroup of the Pediatric Acute Lung Injury and Sepsis Investigator (PALISI) Metwork

Supplemental Materials

Table S1: Contributing centers

Center	City	State
Riley Hospital for Children, Indiana University School of Medicine	Indianapolis	IN
Children's Hospital of Philadelphia, University of Pennsylvania Perelman School of Medicine	Philadelphia	PA
St. Jude's Children's Research Hospital	Memphis	TN
Benioff Children's Hospital, University of California San Francisco	San Francisco	CA
Children's National, George Washington University School of Medicine and Health Sciences	Washington DC	DC
Phoenix Children's Hospital	Phoenix	AZ
Nationwide Children's Hospital, The Ohio State University	Columbus	OH
University of Texas at MD Anderson Cancer Center	Houston	TX
Duke Children's Hospital, Duke University	Durham	NC
Children's Healthcare of Atlanta, Emory University	Atlanta	GA
Masonic Children's Hospital, University of Minnesota	Minneapolis	MD

Table S2: Sensitivity analyses for age groups to identify respiratory rate optimal cutpoint at 4 hours

Age groupings	Optimal respiratory rate (breaths per minute) Chosen by Youden's Index
5 age groups	
1. Under 1 year	42
2. 1-3 years	36
3. 3-5 years	35
4. 5-10 years	35
5. Over 10 years	37
3 age groups	
1. Under 1 year	42
2. 1-10 years	35
3. Over 10 years	37
2 age groups	
1. Under 10 years	36
2. Over 10 years	37
Entire cohort	38

Table S3: Specific diagnosis leading to transplant assessed by NIPPV failure status

	Cohort n=153	Failed n=97	Success n=56
Acute lymphoblastic leukemias (n=31)			
ALL	29 (19%)	20 (21%)	9 (16%)
Biphenotypic leukemia	2 (1%)	1 (1%)	1 (2%)
Myeloid leukemias (n=38)			
AML	35 (22%)	22 (23%)	13 (23%)
CML	1 (0.5%)	0 (0%)	1 (2%)
JMML	2 (1%)	1 (1%)	1 (2%)
MDS	7 (5%)	6 (6%)	1 (2%)
MPS	1 (0.5%)	1 (1%)	0 (0%)
Immunodeficiencies (n=14)			
SCID	4 (3%)	1 (1%)	3 (5%)
Various genetic induced immunodeficiencies	6 (4%)	4 (4%)	2 (4%)
Unspecified	6 (4%)	5 (5%)	1 (2%)
Hemoglobinopathies (n=11)			
Sickle cell disease	9 (6%)	4 (4%)	5 (9%)
Thalasemias	2 (1%)	0 (0%)	2 (4%)
Bone Marrow failure (n=15)			
Diamond Blackfan Anemia	3 (2%)	2 (2%)	1 (2%)
Aplastic Anemia	4 (3%)	2 (2%)	2 (4%)
Fanconi's Anemia	4 (3%)	2 (2%)	2 (4%)
Shwachman diamond syndrome	1 (0.5%)	1 (1%)	0 (0%)
Congenital amegakaryocytic thrombocytopenia	1 (0.5%)	0 (0%)	1 (2%)
Congenital neutropenia	1 (0.5%)	1 (1%)	0 (0%)
Unspecified	1 (0.5%)	1 (1%)	0 (0%)
Metabolic (n=27)			
Osteopetrosis	5 (3%)	3 (3%)	2 (4%)
Krabbe	2 (1%)	1 (1%)	1 (2%)
Metachromatic leukodystrophy	1 (0.5%)	1 (1%)	0 (0%)
Hurlers	4 (3%)	3 (3%)	1 (2%)
Adrenoleukodystrophy	4 (3%)	3 (3%)	1 (2%)
Cartlilage hair hypoplasia	1 (0.5%)	1 (1%)	0 (0%)
Harlor Syndrome	1 (0.5%)	1 (1%)	0 (0%)
Wiskott-Aldrich Syndrome	2 (1%)	1 (1%)	1 (2%)
Epidermolysis Bullosa	3 (2%)	2 (2%)	1 (2%)
Fucosidosis	1 (0.5%)	1 (1%)	0 (0%)
Pelizaeus-Merzbacher Disease	1 (0.5%)	0 (0%)	1 (2%)
Glycogen storage disease type 1B	1 (0.5%)	0 (0%)	1 (2%)
HLH	1 (0.5%)	1 (1%)	0 (0%)
Lymphoma (n=4)			
NK cell lymphoma	1 (0.5%)	1 (1%)	0 (0%)
Burkitt's Lymphoma	1 (0.5%)	1 (1%)	0 (0%)
Mediastinal Gray Zone Lymphoma	1 (0.5%)	1 (1%)	0 (0%)
Mycosis Fungoides/Systemic Lymphoma	1 (0.5%)	0 (0%)	1 (2%)
Unspecified malignant disorders	2 (1%)	1 (1%)	1 (2%)
Unspecified nonmalignant disorder	1 (0.5%)	1 (1%)	0 (0%)

Data is displayed as counts and (percentages). Percentage were rounded to the nearest whole number and therefore totals may not equal 100.

Table S4: Vital signs and respiratory parameters comparing those who were successfully treated with NIV to those who failed NIV.

	Initiation	Hour 2	Hour 4	Hour 8	Hour 12
**Heart Rate (beats per minute)**					
Success	136 (11,157)	127 (113, 147)	126 (108, 143)	127 (105, 141)	117 (100, 130)
Failure	126 (114, 156)	135 (118, 150)	131 (111, 149)	124 (114, 141)	130 (113, 147)
p value	0.970	0.200	0.273	0.915	0.18
**Mean arterial pressure (mmHg)**					
Success	80 (72, 85)	75 (65, 88)	75 (67, 86)	81 (66, 86)	79 (68, 88)
Failure	80 (69, 94)	82 (69, 92)	84 (70, 95)	82 (73, 92)	82 (66, 98)
p value	0.605	0.258	0.093	0.143	0.247
**Respiratory Rate (breaths per minute)**					
Success	35 (26, 44)	31 (26, 42)	31 (24, 37)	33 (22, 37)	32 (24, 40)
Failure	40 (32, 52)	38 (26, 51)	39 (26, 48)	37 (29, 47)	39 (30, 48)
p value	0.030	0.045	0.005	0.003	0.002
**Pulse Oximetry (%)**					
Success	97 (94, 100)	98 (96, 100)	99 (95, 100)	98 (96, 100)	99 (96, 100)
Failure	98 (95, 100)	98 (95, 100)	98 (96, 100)	98 (96, 100)	98 (94, 100)
p value	0.404	0.908	0.894	0.609	0.199
**FiO_2**					
Success	0.50 (0.40, 0.75)	0.43 (0.40, 0.60)	0.40 (0.36, 0.60)	0.40 (0.35, 0.50)	0.40 (0.30, 0.50)
Failure	0.50 (0.40, 0.66)	0.50 (0.40, 0.60)	0.45 (0.35, 0.55)	0.43 (0.35, 0.60)	0.40 (0.35, 0.60)
p value	0.796	0.225	0.950	0.630	0.600
**Inspiratory positive airway pressure (cmH_20)**					
Success	12 (7, 14)	12 (8, 14)	12 (8, 15)	12 (7, 16)	14 (8, 16)
Failure	10 (6, 12)	11 (8, 14)	11 (8, 14)	12 (8, 15)	10 (7, 15)
p value	0.118	0.303	0.263	0.590	0.467
**Expiratory positive airway pressure (cmH_20)**					
Success	6 (6, 8)	8 (6, 8)	8 (6, 8)	8 (6, 8)	8 (6, 8)
Failure	6 (6, 8)	6 (6, 8)	6 (6, 8)	6 (6, 8)	6 (6, 8)
p value	0.848	0.361	0.128	0.200	0.286

Footnote: Data presented are medians with (interquartile ranges) and were compared using a Wilcoxon rank sum test.

Table S5: Univariate regression models for risk of NIPPV failure

	Unadjusted OR, (95% CI)	*p*
Age (years)	0.97 (95% CI: 0.92, 1.03)	0.303
Days on Supplemental O_2 prior to NIPPV	1.1 (95% CI: 1.0, 1.2)	0.038
Vasoactive use prior to or same day as NIPPV initiation	3.7 (95% CI: 1.7, 8.1)	0.001
FiO_2 at start (%)	1.2 (95% CI: 0.3, 4.9)	0.887
FiO_2 at 4 hours	0.4 (95% CI: 0.1, 2.7)	0.377
≥ 10% weight gain	1.5 (95% CI: 0.7, 3.1)	0.301
≥ 15% weight gain	0.8 (95% CI: 0.4, 2.0)	0.700
Renal replacement therapy prior to NIPPV failure	2.7 (95% CI: 0.9, 8.5)	0.088

Footnote: age is presented in years and left as a continuous variable. FiO_2 is presented in percentage. Age, Days on supplemental O2, and FiO2 are presented as a continuous variables. Percent weight gain was calculated by [(weight (kg) at NIV initiation – weight at hospital admission)/weight at hospital admission] x 100.

Table S6: Multivariate model for odds of NIPPV failure with respiratory rate as a continuous variable

	Adjusted OR (95% CI)	*p*
Age (years)	1.0 (95% CI: 0.93, 1.1)	0.969
Days on supplemental O_2 prior to NIPPV	1.1 (95% CI: 0.99, 1.2)	0.099
Respiratory rate at 4 hours	1.1 (95% CI: 1.02, 1.1)	0.002
Vasoactive use	4.6 (95% CI: 1.7, 12.1)	0.002
Matched related donor vs others	0.3 (95% CI: 0.1, 0.9)	0.039

Table S7: Unadjusted and multivariate model for NIPPV failure using 30 breaths per minute at 4 hours as the cutpoint.

	**Odds Ratio (95% confidence interval)**	**p**
**Unadjusted Model**		
Respiratory rate > 30 bpm at 4 hours	3.03 (1.29, 7.07)	0.011
**Multivariable model**		
Respiratory rate > 30 bpm at 4 hours	3.03 (1.29, 7.07)	0.011
Vasoactive age use	4.25 (1.63, 11.06)	0.003
Match related donor	0.33 (0.11, 1.01)	0.052
Days on supplemental O2 prior to NIPPV	1.07 (0.99, 1.16)	0.113
Age	0.99 (0.93, 1.16)	0.836

Table S8: Multivariate model for odds of NIPPV failure with respiratory rate as a continuous variable at 2 hours

Variable	Odds Ratio (95% confidence interval)	p
Respiratory rate at 2 hours	1.03 (1.002, 1.063)	0.035
Vasoactive age use	3.79 (1.55, 9.28)	0.004
Match related donor	0.39 (0.14, 1.07)	0.068
Days on supplemental O2 prior to NIPPV	1.07 (0.99, 1.15)	0.087
Age	1.01 (0.94, 1.08)	0.882

Table S9: Complications of NIPPV assessed by failure or successful use of NIPPV

	Failure n=97	Success n=56
Cardiopulmonary resuscitation*	14 (14%)	0 (0%)
Skin breakdown	5 (5%)	2 (4%)
Epistaxis	0 (0%)	1 (2%)
Emesis without aspiration	2 (2%)	0 (0%)
Aspiration	0 (0%)	0 (0%)
Sedation/Intolerance	5 (5%)	3 (5%)
Excessive coughing	1 (1%)	0 (0%)

*Cardiopulmonary resuscitation during or prior to intubation

Supplemental Figure legends

Supplemental Figure 1. Time to intubation or arrest for those that failed noninvasive positive pressure ventilation

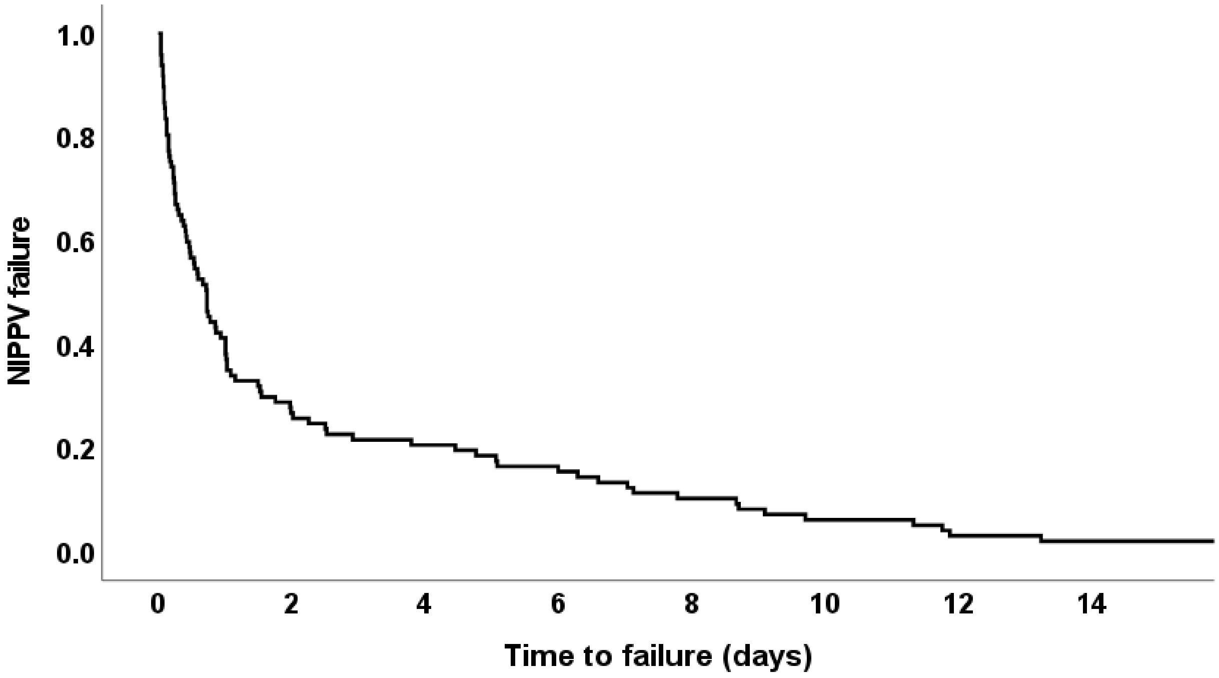

Isolating those that failed noninvasive, the present of failure of represented on the y axis with time to failure in days represented on the x axis. While there was a range of time to failure of noninvasive positive pressure ventilation, the majority of patients failed within the first two days.

Supplemental Figure 2: Median respiratory rate trends over time stratified by NIPPV success and failure

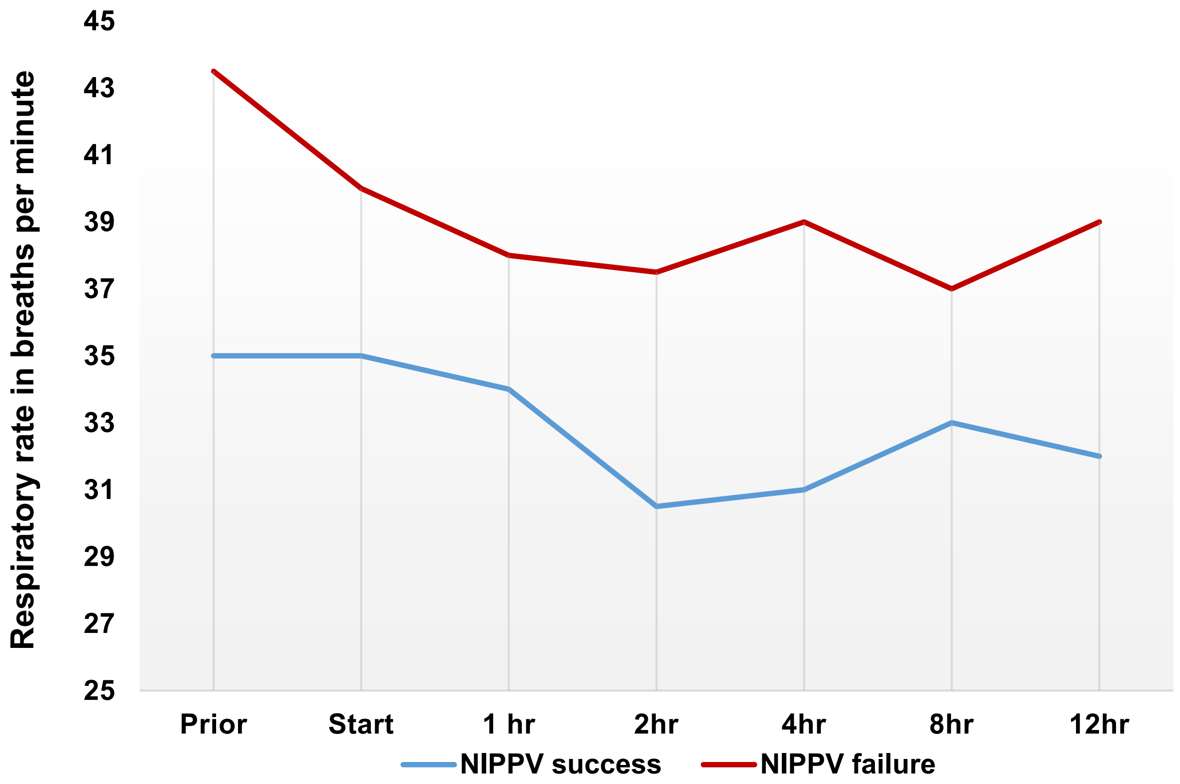

Median respiratory rate in beats per minute is trended over the first 12 hours of noninvasive positive pressure ventilation (NIPPV) use and separated by success or failure of NIPPV. Those who failed noninvasive consistently had a higher median respiratory rate at all time points.

Supplemental Figure 3: Distribution of respiratory rate assessed by successful use of NIV at hour 4 of NIV.

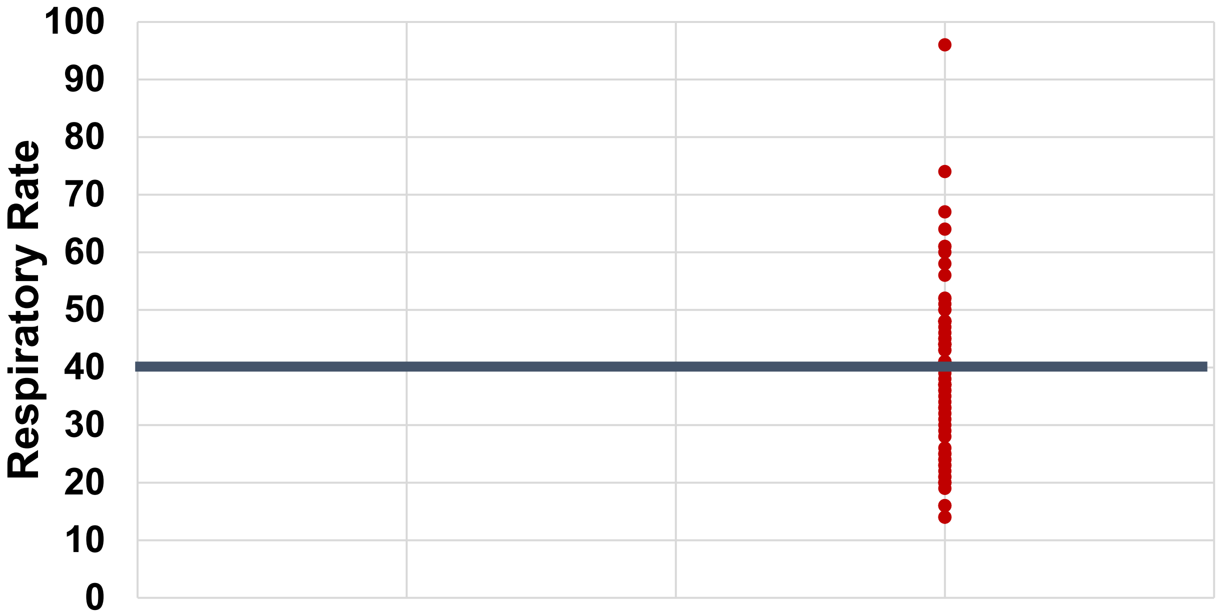

Variation in respiratory rate at hour 4 of noninvasive demonstrates that those who failed had a higher proportion of patients with a respiratory rate over 40 breaths per minute.
